# Evaluation of Cerebral Microvascular Regulatory Mechanisms with Transcranial Doppler in Fabry Disease

**DOI:** 10.3390/brainsci10080528

**Published:** 2020-08-07

**Authors:** Pedro Castro, Mariana Gutierres, Gilberto Pereira, Susana Ferreira, João Paulo Oliveira, Elsa Azevedo

**Affiliations:** 1 Department of Clinical Neurosciences and Mental Health, Faculty of Medicine of University of Porto, 4200-319 Porto, Portugal; pedromacc@med.up.pt (P.C.); marianagutierres96@hotmail.com (M.G.); 2 Cardiovascular Research and Development Unit, Faculty of Medicine of University of Porto, 4200-319 Porto, Portugal; 3 Department of Neurology, Centro Hospitalar Universitário de São João, 4200-319 Porto, Portugal; gilbertofpp@gmail.com; 4 Department of Medical Genetics, Faculty of Medicine of University of Porto and Centro Hospitalar Universitário de São João, 4200-319 Porto, Portugal; jgsusana@gmail.com (S.F.); jpo@med.up.pt (J.P.O.); 5 i3S–Instituto de Investigação e Inovação em Saúde, 4200-135 Porto, Portugal

**Keywords:** cerebral blood flow, neurovascular coupling, Fabry disease, small vessel disease, transcranial Doppler

## Abstract

Background: Fabry disease (FD) causes cerebrovascular disease (CVD) even if asymptomatic, and this is why it is important to identify non-invasive methods to monitor the disease. We evaluated the usefulness of the cerebral autoregulation, vasoreactivity, and neurovascular coupling assessed by transcranial Doppler (TCD) in FD. Methods: Ten adult patients with classic phenotype FD, without clinical expression of CVD, and ten healthy controls, were included. We monitored cerebral blood flow velocity with TCD in the middle and posterior cerebral arteries, blood pressure, heart rate, and non-invasive expired carbon dioxide (CO_2_). Cerebral autoregulation was calculated from the spontaneous oscillations of blood pressure, cerebral vasoreactivity through CO_2_ inhalation and hyperventilation and neurovascular coupling by the flow velocity change to visual stimulation. Results: FD male patients showed blunted vasoreactivity in posterior circulation (0.70 ± 0.36%/mmHg vs. 1.09 ± 0.18%/mmHg CO_2_, *p* = 0.01) and impaired neurovascular coupling (overshoot 15 ± 2.9% vs. 28 ± 6.1%, *p* < 0.01). Cerebral autoregulation was similar to controls. Conclusion: Male patients with FD classic phenotype and hitherto clinical expression of CVD already show impairment of cerebral vasoreactivity and neurovascular coupling. It supports the notion of an early dysfunction of cerebral microvascular in a presymptomatic stage of CVD in FD and that TCD could be useful in its assessment.

## 1. Introduction

Fabry disease (FD) is a rare X-linked glycosphingolipidosis caused by pathogenic alleles of the gene encoding the lysosomal enzyme alpha-galactosidase (*GLA*; OMIM*300644). The resultant enzyme deficiency leads to the systemic accumulation of globotriaosylceramide (Gb3), primarily in vascular endothelium and smooth muscle cells [1].

Stroke and transient ischemic attacks are common neurological complications of FD in young adults [1]. This might occur before FD diagnosis and in the absence of other systemic clinical manifestations [2]. Brain magnetic resonance imaging (MRI), is well established in the evaluation of FD [1]. Some conspicuous findings are a dilative vasculopathy of the vertebrobasilar arteries characterized by elongation, tortuosity, ectasia, and focal aneurisms [3,4]. Increased non-atherosclerotic carotid intima-media thickness [3,4] and impaired brachial flow-mediated dilatation [4] are additional aspects of the large-sized arteriopathy. 

However, cerebrovascular events in FD are not caused by large artery pathology but are predominantly due to small vessel ischemic disease [1]. The extensive white matter lesions found in the brain are strong evidence of the underlying cerebral microvascular disease [5]. Nevertheless, these radiological markers do not inform about the functional status of the microvessels. It has been suggested that cerebral microvascular dysregulation might be present in asymptomatic FD patients [3]. Therefore, there is a need to identify non-invasive methods to effectively evaluate early this vasomotor disturbance and monitor its progression. 

More so, enzyme replacement therapy (ERT) with α-galactosidase is available [6,7], and there is some evidence that it exerts beneficial effects on the cerebral blood flow [8], which may prevent stroke [9,10] and other complications [11]. An early diagnosis and careful monitoring is extremely important for treatment decisions and prevention of organ dysfunction.

The transcranial Doppler (TCD) is a non-invasive technique routinely used to measure cerebral blood flow velocity (CBFV) [12]. Notably, it can also quantify the performance of the cerebrovascular regulation occurring at small vessels [9]. These are cerebral autoregulation (CA), the adaption to systemic fluctuations in blood pressure [13], vasoreactivity (VR) to CO_2_ [2,14] and neurovascular coupling (NVC), the functional hyperemia following neuronal activation [3,15]. Studies on the cerebrovascular regulation in FD are not extensively reported. VR tests with acetazolamide [8] were suggestive of an abnormal vasodilation but requires the injection of a pharmacological agent, not with insignificant side effects. Despite the fact that worse CA is linked to unfavorable outcomes in many cerebrovascular disorders [16], including in small-vessel ischemic strokes, it has not been studied in FD with updated standards [13]. Finally, NVC can be altered in some FD patients [2], but more work is needed to corroborate the potential use of TCD. 

We aimed to study the cerebral microvascular regulation (CA, VR to CO_2_ and NVC) by TCD in patients with classic FD, in order to identify useful markers for patient assessment and disease monitoring.

## 2. Materials and Methods

### 2.1. Participants

All patients ≥18 years-old with a genetic diagnosis of classic FD [1], without stroke/transient ischemic attack, and followed at our center were invited to participate. Our center is a national referral site for FD patient’s evaluation and treatment. This study was conducted in accordance with the Declaration of Helsinki and the research protocol was approved by the institutional Health Ethics Committee (CES 180/19, June 2019). A written informed consent was obtained from all the participants. Exclusion criteria were absence of bone window for TCD, significant cervical (≥50% by the North-American symptomatic carotid endarterectomy trial method) or intracranial (≥50% by Baumgartner’s hemodynamic criteria) arterial stenosis, previous cerebrovascular disease, severe respiratory pathology, and refusal to sign the consent. Healthy controls were recruited among the hospital/university staff matched for patients’ gender and age.

### 2.2. Clinical Examination and Baseline Assessment 

Medical history, medication, and laboratorial analysis were retrieved from the medical records. Body-mass index was calculated. Systolic and diastolic blood pressure (BP) was measured by sphygmomanometer. Orthostatic hypotension was diagnosed if there was a decrease in systolic BP of 20 mmHg or diastolic BP of 10 mmHg [17]. Cognitive performance was assessed with the Montreal Cognitive Assessment (MoCA). 

### 2.3. Cervical and Transcranial Ultrasound

Cervical and transcranial ultrasounds were performed with a Philips Healthcare iU22 device (Eindhoven, The Netherlands). In order to identify hemodynamically significant stenosis, we evaluated morphological and hemodynamic parameters of the cervical common, external and internal carotid arteries, as well as of the vertebral arteries, and hemodynamic parameters of the main intracranial arteries—middle, anterior and posterior cerebral arteries, intracranial vertebral and basilar arteries.

### 2.4. Monitoring Protocol with Transcranial Doppler 

Participants were monitored with TCD (Doppler Box X, DWL, Germany), with 2-MHz probes mounted on a headband to record CBFV in M1 segment of the right middle cerebral artery (MCA) and P2 segment of the left posterior cerebral artery (PCA); beat-by-beat arterial BP was recorded with Finometer (FMS, Amsterdam, The Netherlands), heart rate (HR) by electrocardiogram, and end-tidal CO_2_ (EtCO_2_) by non-invasive capnography (Respsense Nonin, Amsterdam, The Netherlands). Data were synchronized and digitally recorded at 400 Hz with Powerlab (AD Instruments, Oxford, UK) for offline analysis.

#### 2.4.1. Cerebral Autoregulation

A resting supine 5-min segment of data was used to assess CA by the transfer function from the spontaneous oscillations from BP to CBFV in frequency domain accordingly to proposed standards [13]. The main parameters are gain and phase. Lower gain and higher phase indicate a more effective CA. Values were reported in the very-low (VLF: 0.02–0.05 Hz) and low (LF: 0.05–0.20 Hz) frequency bands [13]. 

#### 2.4.2. Cerebral Vasoreactivity

Patients were monitored in the supine position, (i) at rest, in ambient air, (ii) after inhalation of a 5% CO_2_ + 95% O_2_ mixture for two minutes, and after recovery (iii) two minutes of non-forced hyperventilation. A step change in EtCO_2_ of 7–10 mmHg was guaranteed for hyper/hypocapnia. VR is calculated by the slope of the relation of the averaged values of mean CBFV and EtCO_2_ for each stage [14]. We also calculated the cerebrovascular vasoreactivity index (as mean BP/mean CBFV) and expressed VR as slope of the relation of the averaged values of mean CVR and EtCO_2_.

#### 2.4.3. Neurovascular Coupling

The protocol consisted in seven cycles each comprising in a resting phase of 20 s (closed eyes) and a stimulation phase of 40 s looking at the projection of a checkerboard pattern [18]. Cycles were synchronized and averaged. NVC is quantified as the maximum change of the CBFV during stimulation ($\frac{maximum\;CBFV-baseline\;CBFV}{baseline\;CBFV}\times 100\%$) [19].

### 2.5. Statistical Analysis

Normality of continuous variables were checked by Shapiro-Wilk test. Baseline variables were compared by student’s *t*- and Chi-square tests. Cerebrovascular tests were compared between groups (FD vs. control), with repeated-measures ANOVA with arterial territory (MCA vs. PCA) and gender as factors. Bonferroni correction was used to control for multiple comparisons. The association of cerebrovascular tests results and MoCA scores was determined by Spearman’s correlation. All statistical calculations were made with the SPSS v26.0 software (SPSS® Inc., Chicago, IL, USA). *p* < 0.05 was considered statistically significant.

## 3. Results

Ten FD patients and 10 matched controls completed the protocol. The patients were hemizygous (males, *n* = 4) or heterozygous (females, *n* = 6) for the following deleterious *GLA* gene variants, associated with typical manifestations of the classic phenotype [1]: c. (195–1193_640–560dup) *n* = 3; p. (Arg220Ter) *n* = 2; p. (Cys94Ser) *n* = 2; p. (Ser148Asn) *n* = 2 and p. (Trp287Leu) *n* = 1.

The demographic and anthropometric characteristics did not differ between patients and controls (Table 1). All 4 FD males and 2 of the FD females were receiving ERT, respectively for an average of 14 ± 2 years and 6 ± 6 years. None of the patients with FD exhibited orthostatic hypotension. MoCA scores in FD were within normal range for age and education [20]. HR was lower in FD patients as compared to controls (*p* = 0.02).

**Figure 1:**
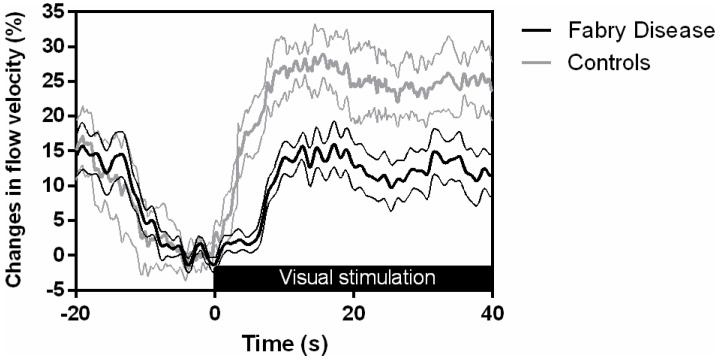
Group-averaged evoked systolic cerebral blood flow velocity (CBFV) responses during visual stimulation with flickering checkerboard. Male healthy controls are represented by thick lines and male Fabry disease patients by black lines. Thinner lines represent standard deviation.

The results of the TCD tests are reported in Table 2. Significant findings were that in FD male patients as compared to male controls (i) VR to CO_2_ was reduced (0.70 ± 0.36%/mmHg vs. 1.09 ± 0.18%/mmHg CO_2_, *p* = 0.01); and (ii) CBFV showed a smaller increase during visual stimulation (systolic overshoot 15 ± 2.9% vs. 28 ± 6.1%, *p* < 0.01 and mean overshoot 16 ± 5.8% vs. 32 ± 6.2%, *p* < 0.01). The differences in NVC responses can be inspected graphically in Figure 1. CA was similar between groups. 

Cognitive performance measured with MoCA was not correlated with NVC or VR in PCA (Table 3). Also, HR was not correlated with NVC (systolic overshoot *r* = 0.21, *p* = 0.38; mean overshoot *r* = – 0.10, *p* = 0.67) or VR to CO_2_ in PCA (*r* = 0.09, *p* = 0.73).

## 4. Discussion

In this case-control study, comparing 4 males and 6 females carrying GLA gene variants associated with the classic phenotype of FD, without previous history of cerebrovascular events, and 10 appropriately matched healthy subjects, significant impairment of VR to CO_2_ and a disturbed NVC, both in the PCA territory, were observed in male patients. These differences suggest that classically affected FD males have dysfunction of the brain small arteries since the early stages of disease. A plausible explanation for the normalcy of those parameters in FD female is their heterozygous genetic condition, as opposed to the hemizygous status of the affected males. 

### 4.1. Posterior Circulation Microvascular Impairment in Males with Long-Term ERT

The impaired cerebral VR to CO_2_ in the PCA and visual cortex NVC, observed in our cohort of males with classical FD with no history of stroke transient ischemic attacks, despite its small size, is in line with a variety of data showing that the posterior circulation is more vulnerable in FD [5,21]. However, when we measured VR taking into account the MAP changes during the test, as expressed by the variations on CVR, no significant differences were observed. Therefore, the changes in CBFV during CO_2_ challenge might be influenced by concomitant changes in BP. Thus, we cannot exclude that disautonomia of FD could affect our VR results. Nevertheless, previous studies document impairment of VR in FD [5,22]. The reason for the higher vulnerability of the posterior circulation is not known [5]. A tendency for reduced cerebral VR in the MCA during hypercapnia (apnea) has been suggested [22] which improved with ERT [23]. Since our cohort is mainly on ERT, this may justify why our patients had normal VR in anterior circulation. Differently, our study highlights the fact that PCA territory continues to show abnormalities despite long-term ERT. A previous study using acetazolamide found an altered VR in PCA that reversed after therapy [8]. Comparison with our results may be difficult since they used positron emission tomography and it should be noted that the mechanisms that mediate the effects of CO_2_ and of acetazolamide on the vascular wall may not be entirely overlapping. In the mentioned study, ERT was administered for 6 months only, which should be enough to promote an effective and rapid clearance of Gb_3_ from the vascular endothelium [6,7,10]. Based on our cohort of long-term treatment, it appears that the ERT effect could be transient in posterior microvasculature and that the underlying mechanism is not only endothelial dysfunction. 

The maximal CBFV increase during visual stimulation was significantly lower in FD males compared to control males, reflecting a worse cerebrovascular response to visual activation tasks. These results indicate that NVC in the visual cortex is impaired in adult males with classic FD, lending further support to the hypothesis that the posterior circulation is more vulnerable to the vasculopathy of FD. A disturbed neurovascular coupling in FD have already been reported by our group in a previous study with functional transcranial Doppler [3]. Impaired NVC in the occipital cortex has also been described in individuals with other hereditary small vessels diseases, such as CADASIL [24]. Although it is plausible that these TCD results are pathophysiologically related to endothelial dysfunction, the role of the autonomic nervous system in the cerebral regulation cannot be ignored, as shown in other diseases associated with autonomic failure [25]. It should be noted that none of our FD patients exhibited orthostatic hypotension or any other major manifestations of dysautonomia. We previously report, NVC impairment in a different cohort of FD patients. Compared with the previous cohort, our patients were older and with longer time on ERT. This study confirms that NVC is impaired in FD patients, despite older age and longer ERT than the previous cohort. Moreover, our study advanced that vasomotor deregulation seems to be more pronounced in posterior circulation, as VR is probably also affected in PCA, and not the result of a more general cerebrovascular disturbance, reflected by a preserved CA. In a study of 22 young adult males with FD, naïve to ERT, Hilz et al. [26] observed an some impairment of CA based on transfer function gain between BP and CBFV oscillations but not phase, the most robust CA parameter [13]. It is also possible that ERT might improve CA overtime like the effect shown on the VR in MCA.

In FD patients, we found no association between NVC or VR to CO_2_ in PCA parameters and MoCA scores. This supports the notion the altered cerebrovascular regulation comes before neurological manifestations. If these TCD indexes represent early markers of brain damage only further prospective work will provide the answer. In agreement with the observation that bradycardia is one of the first signs of cardiac involvement in FD [27], resting HR was significantly lower in our patients than in the controls. However, this finding does not seem to have an impact upon VR and NVC results [14] and were not correlated.

### 4.2. Pathophysiological Considerations

MRI has been routinely used in the follow-up of FD [5]. While white-matter hyperintensities and increased basilar artery diameter [5,21] are the most commonly reported abnormalities, the specificity of the “pulvinar sign”—an increased pulvinar signal intensity on MRI T1-weighted imaging—is a matter of debate [5,28]. 

Although the underlying pathophysiology is still unclear, the pathological hallmark of the vasculopathy of FD is the accumulation of Gb_3_ in the cerebral vascular endothelium and smooth muscle cells [1]. The increased IMT [3,4] and an impaired FMD [4] that have been observed in FD patients indicate that large arteries are dysfunctional as well, perhaps because of the smooth muscle cell involvement, and that the FD vasculopathy is not circumscribed to the microvasculature. Increased circulating levels of endothelial biomarkers, such as sVCAM-1 and TNF-α, are suggestive of endothelial dysfunction [22], and there is also evidence of a prothrombotic state [29] and of dysregulation of the nitric oxide pathway [30]. Currently available data suggest that ERT has an overall beneficial effect on the long-term natural history of FD [10,11] preventing stroke recurrence [9,10] and improving other complications. ERT for FD has already been shown to improve cerebral blood flow dynamics as assessed by several different methods, including positron emission tomography [8] and TCD [23], but its impact on the cerebral vasculopathy of FD remains incompletely understood and further TCD cerebrovascular regulation studies might help in clarifying this issue.

## 5. Limitations

The small size of the patient cohort is a major limitation of our study, curtailing its statistical power, but the strict genotypic characterization of the patients as well as the overall homogeneity granted by the single center study design might have counter-balanced the size limitation. Since the statistical significance of our major findings stood true even after adjustment for the multiple comparisons, we believe that they truly reflect a pathophysiological condition of the cerebral posterior arterial territory in males with classical FD, and that type II errors (i.e., having missed real pathophysiological phenomena) might have been more probable. Nevertheless, further studies with larger gender-stratified patient cohorts, in different populations, will be necessary to confirm our results and caution should be taken in the interpretation of the results. 

## 6. Conclusions

In conclusion, male patients with FD and hitherto clinical expression of CVD already show impairment of neurovascular coupling in posterior circulation. It supports the notion of an early dysfunction of cerebral microvasculature in a presymptomatic stage of CVD in FD. Therefore, functional cerebrovascular testing with TCD may be useful to detect early symptoms of CVD and possible therapeutic intervention, although it is still unclear if all the signs detected through TCD can improve with the current available treatments. 

## Figures and Tables

**Table 1:** Baseline characteristics of Fabry disease patients and controls.

Participant Characteristics	Fabry (*n* = 10)	Controls (*n* = 10)	*p* ^a^
Male	4 (40)	4 (40)	
Age at inclusion, years	42 ± 13	41 ± 13	0.91
Age at diagnosis, years	27 ± 14		
Hypertension	2 (20)	0 (0)	
Diabetes Mellitus	1 (10)	0 (0)	
Dyslipidemia	2 (20)	0 (0)	
Tobacco	0 (0)	0 (0)	
BMI	25.0 ± 4.9	23.2 ± 3.2	0.34
MoCA score	22 ± 6		
Chronic Medication			
Statins	2 (20)	0 (0)	
Antiplatelets	1 (10)	0 (0)	
Antihypertensives	5 (50)	0 (0)	
ERT therapy (4 males; 2 females)	6 (60)		
Age of start of ERT therapy, years	30 ± 21		
Laboratorial results			
Total cholesterol, mg/dL	163 ± 20		
LDL cholesterol, mg/dL	91 ± 15		
HDL cholesterol, mg/dL	57 ± 9		
Fasting plasma glucose, mg/dL	88 ± 12		
Systemic Hemodynamics			
Systolic BP, mmHg	117 ± 11	118 ± 15	0.80
Diastolic BP, mmHg	67 ± 11	68 ± 10	0.80
Heart rate, bpm	57 ± 10	69 ± 10	0.02
EtCO_2_, mmHg	39 ± 3	37 ± 4	0.32

BMI: body mass index; BP: blood pressure; ERT: enzyme replacement therapy; EtCO_2_: end-tidal carbon dioxide; HDL/LDL: high/low-density lipoprotein MoCA: Montreal Cognitive Assessment. All values expressed as number (%) or mean ± SD **^a^** *p* value of Student’s T- or Chi-square tests.

**Table 2:** Comparison of the cerebrovascular tests assessed by transcranial Doppler between Fabry disease and control groups, categorized by sex.

	Fabry (*n* = 10)		Controls (*n* = 10)		*p* ^a^
	Male	Female	Male	Female	
**Cerebral Autoregulation**					
MCA VLF Gain, %/mmHg	1.06 ± 0.55	1.13 ± 0.44	0.86 ± 0.39	0.87 ± 0.45	0.58
LF Gain	1.30 ± 0.21	1.85 ± 0.47	1.41 ± 0.46	1.43 ± 0.33	0.40
VLF Phase, radians	0.94 ± 0.51	1.29 ± 0.23	1.16 ± 0.80	0.93 ± 0.14	0.26
LF Phase	0.87 ± 0.37	0.70 ± 0.18	0.95 ± 0.25	0.64 ± 0.20	0.44
PCA VLF Gain, %/mmHg	1.15 ± 0.60	1.23 ± 0.41	1.28 ± 0.80	0.91 ± 0.54	0.35
LF Gain	2.14 ± 0.99	2.02 ± 0.37	1.86 ± 0.56	1.68 ± 0.52	0.83
VLF Phase, radians	0.88 ± 0.53	1.33 ± 0.20	1.16 ± 0.64	0.91 ± 0.44	0.19
LF Phase	0.70 ± 0.10	0.69 ± 0.19	1.06 ± 0.45	0.63 ± 0.23	0.99
**Vasoreactivity to CO_2_**					
MCA, MFV %/mmHg CO_2_	1.87 ± 0.67	1.59 ± 0.56	1.58 ± 0.34	2.05 ± 0.57	0.39
MCA, CVR %/mmHg CO_2_	−2.61 ± 0.42	−3.32 ± 0.89	−2.96 ± 0.67	−2.82 ± 0.97	0.37
PCA, MFV %/mmHg CO_2_	0.70 ± 0.36 *	0.86 ± 0.10	1.09 ± 0.18	1.14 ± 0.31	0.01
PCA, CVR %/mmHg CO_2_	−2.85 ± 0.45	−2.43 ± 0.92	−2.81 ± 0.46	−3.10 ± 0.24	0.79
**Neurovascular coupling**					
Overshoot Systolic CBFV, %	15 ± 2.9 *	20 ± 7.2	28 ± 6.1	20 ± 6.3	< 0.01
Overshoot Mean CBFV, %	15 ± 5.8 *	25 ± 10	32 ± 6.2	27 ± 4.8	< 0.01

CBFV: cerebral blood flow velocity; Hz: hertz; VLF/LF: very-low (0.02–0.05 Hz) and low frequency (0.05–0.2 Hz) bands; MCA: middle cerebral artery; MFV: mean flow velocity; PCA: posterior cerebral artery. All values are in means ± SD. ^a^ *p* value of repeated-measures ANOVA for the interaction between group variable (Fabry vs. heathy controls), sex, and arterial territory (MCA vs. PCA) with significant differences after Bonferroni corrections marked with asterisk (*).

**Table 3:** Correlation between selected cerebrovascular regulation indexes and cognitive performance in Fabry disease patients.

Parameters	MoCA Scores	
Cerebral Vasoreactivity	Spearman’s Coefficient	*p* value
VR to CO_2_ PCA, %/mmHg CO_2_	0.22	0.54
**Neurovascular coupling**		
Overshoot Systolic CBFV, %	−0.60	0.07
Overshoot Mean CBFV, %	−0.48	0.16

CBFV: cerebral blood flow velocity; MoCA: Montreal Cognitive Assessment; PCA: posterior cerebral artery; VR: vasoreactivity.
